# The efficacy and safety of massage therapy as an adjuvant in the treatment of hypoxic ischemic encephalopathy in neonates: A protocol for systematic review and meta-analysis

**DOI:** 10.1097/MD.0000000000028963

**Published:** 2022-04-01

**Authors:** Wang Meizhuang, Haizhu Xu, Youhong Chen

**Affiliations:** a Pediatric Ward 2, Second Affiliated Hospital of Hainan Medical College, Haikou, Hainan Province, China; b Department of Outpatient, Second Affiliated Hospital of Hainan Medical College, Haikou, Hainan Province, China.

**Keywords:** hypoxic ischemic encephalopathy, massage, meta-analysis, neonates, protocol

## Abstract

**Background:**

Neonatal hypoxic-ischemic encephalopathy (HIE) has become a major problem that endangers the life and health of newborns. It is the most serious complication after neonatal asphyxia with a high mortality rate. Even survivors of HIE would suffer permanent neurological developmental impairment that seriously affects the growth and development in the future. Previous studies have shown that massage therapy can improve the prognosis of neonatal HIE. However, the efficacy of massage therapy on the growth, development, and sleep in neonates with HIE reported by various studies is inconsistent, which will be thoroughly assessed in this meta-analysis.

**Methods:**

Randomized controlled trials of massage therapy on the growth, development, and sleep neonates with HIE published before February 2022 will be retrieved from the PubMed, Embase, Web of Science, Cochrane Library, China National Knowledge Infrastructure, Wan Fang Database, Chinese Biomedical Literature Database, VIP Database for Chinese Technical Periodicals, and Clinical Trial Register. Literature selection, data extraction, risk of bias assessment, and meta-analyses will be independently completed by 2 researchers. Meta-analysis will be performed by using RevMan5.4.

**Results:**

The results of this meta-analysis will be submitted to a peer-reviewed journal for publication.

**Conclusion:**

This systematic review provides a high-quality synthesis to assess the effect of massage therapy on growth, development, and sleep in neonates with HIE.

**OSF registration number:**

DOI 10.17605/OSF.IO/G9WXN.

## 1 Introduction

Neonatal hypoxic-ischemic encephalopathy (HIE) has become a major problem endangering the life and health of newborns. Four million newborns die of HIE every year worldwide, of which 23% are related to asphyxia. 7.5% to 57.6% of asphyxiated children in China suffer brain damages at varying degrees, and severe cases even die in the early neonatal period. More seriously, 25% to 30% of survivors of HIE may have cerebral palsy, mental retardation, epilepsy spasticity, ataxia, or other permanent neurological sequelae.^[1]^ Even in developed countries like the Europe and the United States, the death and severe disability rates of neonates with moderate-to-high level of HIE are as high as 53% to 61%.^[2]^

The pathogenesis of neonatal HIE is complex and no ideal treatment is available at present. Only supportive and symptomatic treatment are given to neonates with HIE for a long period, which cannot significantly alleviate brain damages and improve the prognosis. Great efforts are required on the prevention and treatment of neonatal HIE, which are extremely challenging in the current neonatal medical field, thus improving its prognosis.^[3]^

Massage therapy is based on the basic theory of traditional Chinese medicine (TCM), and the practice of meridians and acupuncture points, which physically stimulates the body to maintain the blood flow homeostasis in each organ.^[4,5]^ Clinical research studies have shown the acceptable effect of massage therapy on promoting motor development, sleep quality, and learning memory.^[5–7]^ Conventional interventions usually focus on oxygenation and acidosis correction in neonates with HIE. Nevertheless, physical contact and intimate emotional relationship with affected neonates are less concerned, who usually present clinical symptoms of excitement, agitation, and increased muscle tone. Acupuncture is able to reduce the intracranial pressure and improve the blood and oxygen supply to the brain tissue, thus effectively improving the behavior of neonates with HIE.^[8,9]^

The effect of massage therapy on growth and sleep in neonates with HIE have been widely reported,^[10–16]^ although controversial conclusions are obtained and a systematic evaluation is scant. This study aims to comprehensively analyze relevant randomized controlled trials (RCTs) reporting the effect of massage therapy on growth and sleep in neonates with HIE through the meta-analysis, thus providing evidence-based clinical interventions.

## 2 Methods

### 2.1 Study registration

The protocol of the systematic review has been registered on Open Science Framework, with a registration number of DOI 10.17605/OSF.IO/G9WXN. This meta-analysis protocol is based on the Preferred Reporting Items for Systematic Reviews and Meta-analysis Protocols (PRISMA-P) statement guidelines.^[17]^

### 2.2 Data sources and search strategies

RCTs of massage therapy on the growth, development, and sleep neonates with HIE published before February 2022 will be searched in online databases in the combination of MeSH terms and free words, including PubMed, Embase, Web of Science, Cochrane Library, China National Knowledge Infrastructure, Wan Fang Database, Chinese Biomedical Literature Database, VIP Database for Chinese Technical Periodicals, and Clinical Trial Register. The searching strategy in PubMed was displayed in Table 1, and literature searching in other databases will be modified according to the characteristics of each online database.

**Table 1:** Search strategy of the PubMed.

Number	Search terms
#1	Hypoxia-Ischemia, Brain[MeSH]
#2	Anoxia-Ischemia, Brain[Title/Abstract]
#3	Anoxia-Ischemia, Cerebral[Title/Abstract]
#4	Anoxic-Ischemic Encephalopathy[Title/Abstract]
#5	Brain Anoxia-Ischemia[Title/Abstract]
#6	Brain Hypoxia-Ischemia[Title/Abstract]
#7	Brain Ischemia-Anoxia[Title/Abstract]
#8	Brain Ischemia-Hypoxia[Title/Abstract]
#9	Cerebral Anoxia-Ischemia[Title/Abstract]
#10	Cerebral Hypoxia-Ischemia[Title/Abstract]
#11	Cerebral Ischemia-Anoxia[Title/Abstract]
#12	Cerebral Ischemia-Hypoxia[Title/Abstract]
#13	Hypoxia-Ischemia, Cerebral[Title/Abstract]
#14	Hypoxic-Ischemic Encephalopathy[Title/Abstract]
#15	Ischemia-Anoxia, Brain[Title/Abstract]
#16	Ischemia-Anoxia, Cerebral[Title/Abstract]
#17	Ischemia-Hypoxia, Brain[Title/Abstract]
#18	Ischemia-Hypoxia, Cerebral[Title/Abstract]
#19	Ischemic-Hypoxic Encephalopathy[Title/Abstract]
#20	Encephalopathy, Anoxic-Ischemic[Title/Abstract]
#21	Encephalopathy, Hypoxic-Ischemic[Title/Abstract]
#22	Anoxia Ischemia, Brain[Title/Abstract]
#23	Anoxia Ischemia, Cerebral[Title/Abstract]
#24	Anoxia-Ischemias, Brain[Title/Abstract]
#25	Anoxia-Ischemias, Cerebral[Title/Abstract]
#26	Anoxic Ischemic Encephalopathy[Title/Abstract]
#27	Anoxic-Ischemic Encephalopathies[Title/Abstract]
#28	Brain Anoxia Ischemia[Title/Abstract]
#29	Brain Anoxia-Ischemias[Title/Abstract]
#30	Brain Hypoxia Ischemia[Title/Abstract]
#31	Brain Hypoxia-Ischemias[Title/Abstract]
#32	Brain Ischemia Anoxia[Title/Abstract]
#33	Brain Ischemia Hypoxia[Title/Abstract]
#34	Brain Ischemia-Anoxias[Title/Abstract]
#35	Brain Ischemia-Hypoxias[Title/Abstract]
#36	Cerebral Anoxia Ischemia[Title/Abstract]
#37	Cerebral Anoxia-Ischemias[Title/Abstract]
#38	Cerebral Hypoxia Ischemia[Title/Abstract]
#39	Cerebral Hypoxia-Ischemias[Title/Abstract]
#40	Cerebral Ischemia Anoxia[Title/Abstract]
#41	Cerebral Ischemia Hypoxia[Title/Abstract]
#42	Cerebral Ischemia-Anoxias[Title/Abstract]
#43	Cerebral Ischemia-Hypoxias[Title/Abstract]
#44	Encephalopathies, Anoxic-Ischemic[Title/Abstract]
#45	Encephalopathies, Hypoxic-Ischemic[Title/Abstract]
#46	Encephalopathies, Ischemic-Hypoxic[Title/Abstract]
#47	Encephalopathy, Anoxic Ischemic[Title/Abstract]
#48	Encephalopathy, Hypoxic Ischemic[Title/Abstract]
#49	Encephalopathy, Ischemic-Hypoxic[Title/Abstract]
#50	Hypoxia Ischemia, Brain[Title/Abstract]
#51	Hypoxia Ischemia, Cerebral[Title/Abstract]
#52	Hypoxia-Ischemias, Brain[Title/Abstract]
#53	Hypoxia-Ischemias, Cerebral[Title/Abstract]
#54	Hypoxic Ischemic Encephalopathy[Title/Abstract]
#55	Hypoxic-Ischemic Encephalopathies[Title/Abstract]
#56	Ischemia Anoxia, Brain[Title/Abstract]
#57	Ischemia Anoxia, Cerebral[Title/Abstract]
#58	Ischemia Hypoxia, Brain[Title/Abstract]
#59	Ischemia Hypoxia, Cerebral[Title/Abstract]
#60	Ischemia-Anoxias, Brain[Title/Abstract]
#61	Ischemia-Anoxias, Cerebral[Title/Abstract]
#62	Ischemia-Hypoxias, Brain[Title/Abstract]
#63	Ischemia-Hypoxias, Cerebral[Title/Abstract]
#64	Ischemic Hypoxic Encephalopathy[Title/Abstract]
#65	Ischemic-Hypoxic Encephalopathies[Title/Abstract]
#66	or/1-65
#67	Infant, Newborn[MeSH]
#68	Neonate[Title/Abstract]
#69	Newborns[Title/Abstract]
#70	Infants, Newborn[Title/Abstract]
#71	Neonates[Title/Abstract]
#72	Newborn[Title/Abstract]
#73	Newborn Infant[Title/Abstract]
#74	Newborn Infants[Title/Abstract]
#75	or/67-74
#76	Massage[MeSH]
#77	Reflexology[Title/Abstract]
#78	Zone Therapy[Title/Abstract]
#79	Bodywork[Title/Abstract]
#80	Craniosacral Massage[Title/Abstract]
#81	Rolfing[Title/Abstract]
#82	Therapy, Zone[Title/Abstract]
#83	Bodyworks[Title/Abstract]
#84	Massage, Craniosacral[Title/Abstract]
#85	Therapies, Zone[Title/Abstract]
#86	Zone Therapies[Title/Abstract]
#87	or/76-86
#88	Randomized Controlled Trials as Topic[MeSH]
#89	Clinical Trials, Randomized[Title/Abstract]
#90	Controlled Clinical Trials, Randomized[Title/Abstract]
#91	Trials, Randomized Clinical[Title/Abstract]
#92	Random∗[Title/Abstract]
#93	or/88-92
#94	#66 and #75 and #87 and #93

### 2.3 Inclusion criteria of eligible literatures

Inclusion criteria of eligible literatures:

1) Participants: Neonates with HIE.
2) Type of study: RCTs of massage therapy on growth, development, and sleep in neonates with HIE.
3) Intervention measures: Routine treatment and nursing in control group and massage therapy additionally given in experimental group.
4) Outcome measures: Neonatal Behavioral Neurological Assessment, Pittsburgh Sleep Quality Index, body mass index, height, and milk intake.

Exclusion criteria:

1) Duplicate publications;
2) Clinical data are unavailable;
3) Reviews, retrospective studies, case reports, case series, and animal experiments.

### 2.4 Data collection and analysis

**Figure 1:**
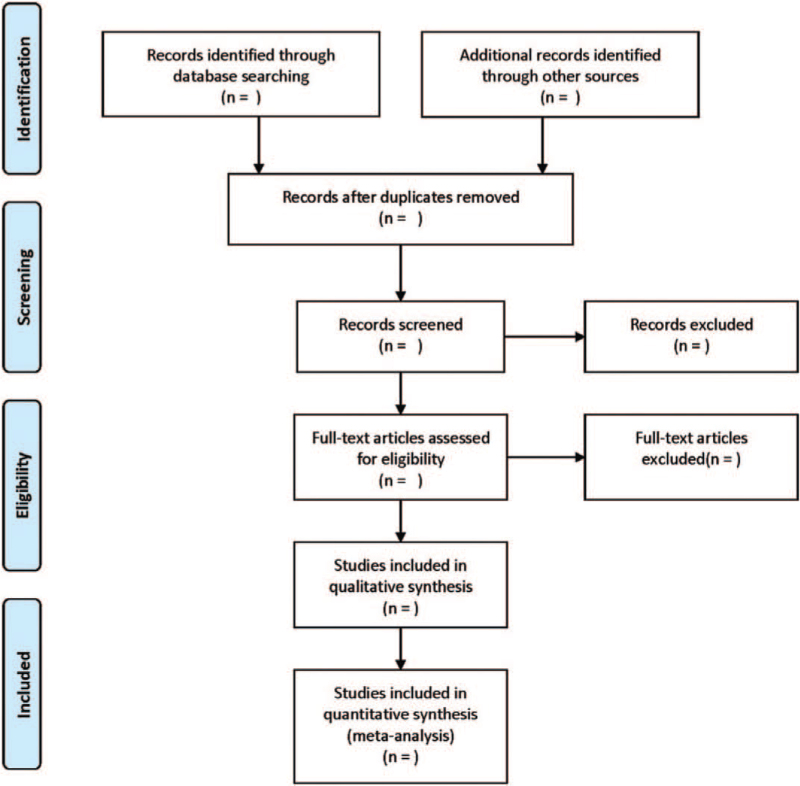
PRISMA flow diagram of the study selection process.

Literature screening and data extraction will be independently performed and double-blind by 2 investigators. Any disagreement will be solved by discussion. Data extraction will be performed according to a pre-designed form, and the following clinical data will be extracted: Basic information of the literature, basic characteristics of neonates with HIE, interventions and control measures, adverse effects, recovery of symptoms, follow-up data, outcome indicators, and details of the quality evaluation of the literature. Missing data will be required for contacting the first author or corresponding author. The flow chart of literature searching and recruitment was demonstrated in Figure 1.

### 2.5 Assessment of the literature quality

In this study, Cochrane Risk Bias Evaluation Tool (version 5.1.0)^[18]^ will be used to evaluate the included RCTs, including whether the randomization is correct, whether the allocation plan is hidden, whether the blinds are complete, whether the data are complete, and whether the results are selectively reported.

### 2.6 Management of missing data

Insufficient or missing data in the literature will be obtained by e-mailing the first or corresponding authors. If still unavailable, only the current available data will be analyzed and the potential impacts will be discussed.

### 2.7 Statistical analysis

RevMan5.4 software (Cochrane Collaboration, London, United Kingdom) will be used for analysis. Continuous variable data will be expressed as standardized mean difference and corresponding 95% confidence interval. I^2^ test will be performed to assess the heterogeneity of studies. *P* > .10 and I^2^ ≤ 50% suggest no heterogeneity and a fixed-effect model will be adopted; Otherwise, a random-effect model will be applied due to the large heterogeneity.

### 2.8 Additional analyses

#### 2.8.1 Subgroup analysis

Subgroup analysis based on factors such as intervention frequency, type of intervention, year, and sample size will be performed.

#### 2.8.2 Sensitivity analysis.

The sensitivity of each index will be analyzed by adopting 1-by-1 elimination method to test the stability of the results.

#### 2.8.3 Assessment of publication bias

If the number of included studies exceeds 10, funnel plots will be drawn for assessing the publication bias.^[19,20]^

### 2.9 Ethical review and informed consent of patients

The contents of this article do not involve moral approval or ethical review and will be presented in print or at relevant conferences.

## 3 Discussion

The severity of HIE is closely related to the degree of asphyxia, and affected neonates usually develop a range of neurological symptoms within 12 hours of birth.^[21,22]^ A significant negative impact of HIE would pose on the growth and development of neonates, and survivors may develop more severe neurological sequelae or even permanent neurological deficits.^[23]^ Therefore, effective nursing interventions following an effective and timely treatment are needed to actively promote the growth and development of neonates with HIE, and improve the prognosis. Massage therapy is a type of early interventions and effective rehabilitations, presenting a good effect on promoting the neurological development of children with brain damages.^[10]^ In this study, we will conduct a meta-analysis of RCTs reporting the effect of massage therapy on growth, development, and sleep in neonates with HIE, aiming to provide evidence-based clinical interventions.

## Author contributions

**Conceptualization:** Youhong Chen, Wang Meizhuang.

**Data curation:** Wang Meizhuang, Haizhu Xu.

**Formal analysis:** Wang Meizhuang.

**Funding acquisition:** Youhong Chen.

**Investigation:** Wang Meizhuang.

**Methodology:** Wang Meizhuang, Haizhu Xu.

**Project administration:** Youhong Chen.

**Resources:** Haizhu Xu.

**Software:** Haizhu Xu.

**Supervision:** Youhong Chen.

**Validation:** Haizhu Xu, Youhong Chen.

**Visualization:** Haizhu Xu.

**Writing – original draft:** Youhong Chen, Wang Meizhuang.

**Writing – review & editing:** Youhong Chen, Wang Meizhuang.
